# Risk Factors for Anastomotic Leakage in Patients with Rectal Tumors Undergoing Anterior Resection within an ERAS Protocol: Results from the Swedish ERAS Database

**DOI:** 10.1007/s00268-021-06054-y

**Published:** 2021-03-17

**Authors:** Daniel Asklid, Olle Ljungqvist, Yin Xu, Ulf O. Gustafsson

**Affiliations:** 1 Department of Clinical Sciences, Division of Surgery, Danderyd Hospital, Karolinska Institute, Stockholm, Sweden; 2 Department of Surgery, Örebro & Institute of Molecular Medicine and Surgery, Örebro University and University Hospital, Karolinska Institute, Stockholm, Sweden; 3 Clinical Epidemiology and Biostatistics, School of Medical Sciences, Örebro University, Örebro, Sweden

## Abstract

**Background:**

Research on risk factors for anastomotic leakage (AL) alone within an Enhanced Recovery After Surgery (ERAS) protocol has not yet been conducted. The aim of this study was to identify risk factors for AL and study short-term outcome after AL in patients operated with anterior resection (AR).

**Methods:**

All prospectively and consecutively recorded patients operated with AR in the Swedish part of the international ERAS® Interactive Audit System (EIAS) between January 2010 and February 2020 were included. The cohort was evaluated regarding risk factors for AL and short-term outcomes, including uni- and multivariate analysis. Pre-, intra- and postoperative compliance to ERAS®Society guidelines was calculated and evaluated.

**Results:**

Altogether 1900 patients were included, 155 (8.2%) with AL and 1745 without AL. Male gender, obesity, peritoneal contamination, year of surgery 2016–2020, duration of primary surgery and age remained significant predictors for AL in multivariate analysis. There was no significant difference in overall pre- and intraoperative compliance to ERAS®Society guidelines between groups. Only preadmission patient education remained as a significant ERAS variable associated with less AL. AL was associated with longer length of stay (LOS), higher morbidity rate and higher rate of reoperations.

**Conclusion:**

Male gender, obesity, peritoneal contamination, duration of surgery, surgery later in study period, age and preadmission patient education were associated with AL in patients operated on with AR. Overall pre- and intraoperative compliance to the ERAS protocol was high in both groups and not associated with AL.

## Introduction

Anastomotic leaks (AL) are detrimental to patients operated with rectal resection, conferring a large burden of morbidity, mortality and worse long-term oncological outcome [1–7]. Leak rates vary from 5 to 20%, the highest rates reported in procedures involving total mesorectal excision (TME) [2, 3]. The list of risk factors is wide and sometimes contradictory [2, 3, 8–11].

ERAS programs reduce surgical stress resulting in faster recovery, reduced postoperative morbidity/complications and shorter LOS and are gaining popularity worldwide [12–14]. Reports on potential associations between specific perioperative care items or compliance to the ERAS®Society guideline protocol and AL specifically within an ERAS protocol have not yet been published.

Our main hypothesis was that compliance to the ERAS protocol, overall or in single intervention variables, has an effect on AL in patients operated with AR. The aim of the current study was to identify risk factors for AL, investigate the significance of compliance to the ERAS protocol in relation to AL, and to study outcome from surgery in AL and non-AL patients operated with AR due to rectal tumor within the Swedish part of the international ERAS database.

## Methods

### Study design and setting

This retrospective multicenter cohort study aimed to investigate potential predictors for AL in patients with rectal tumor (benign or malignant) operated with anterior resection. Compliance to the ERAS protocol and short-term outcome for patients with AL were also investigated. The ERAS®Society guideline for colorectal surgery consists of 25 evidence-based interventions [13] and all centers aim to treat their patients in agreement with this protocol. The international ERAS®Society Interactive Audit System (EIAS) database (EIAS) [15] was used as the source of data. EIAS assembles more than 90 000 consecutive patients records, each with more than 300 variables and includes information on compliance to ERAS guidelines [13]. The Swedish part of the international ERAS database was validated in 2020 with excellent results regarding coverage, missing values and accuracy of data and used for the current study (submitted for publication).

Data on AL, perioperative variables, compliance to the ERAS protocol and outcome from surgery were collected from the EIAS database between January 1, 2010, and February 27, 2020. The definition of AL in EIAS is based on radiological diagnosis or intervention and/or reoperation.

The criteria set out in the Strengthening the Reporting of Observational studies in Epidemiology (STROBE) checklist was met conducting this publication. The study was approved by the Regional Ethical Review Board in Stockholm (2020–00,435).

### Participants

All consecutive patients with benign or malignant rectal tumor (*N*=1900) operated with open, laparoscopic or robotic AR and prospectively registered in the database were included in the study. Emergency surgery was not included. Rectal tumor was defined as a lesion within 15 cm from anal verge. All tumor stages were included, and patients were analyzed according to intention-to-treat.

### Outcome variables

Primary outcome was AL within 30 days from primary surgery. Secondary outcomes were possible consequences of AL: 30-day complications (Clavien I–II, ≥ III)[16], reoperation, LOS and death (30-day).

### Exposure variables

Depending on availability of data in EIAS and results from prior research, the following variables were retained as potential risk factors in multivariate analysis based on purposeful selection modeling approach [17]: gender, age, body mass index, peritoneal soiling, preoperative radiotherapy, year of surgery, duration of primary surgery and preadmission patient education.

### Data analysis

No formal sample size calculation was conducted since secondary data were used, but it is feasible to consider power to detect a meaningful effect. At the 5% significance level, the sample size allows for the detection of an odds ratio > 1.7 for a binary predictor variable with 80% power [18].

Both unadjusted and adjusted models were performed. Chi-squared test or Fisher exact test (for categorical variables with cell size less than 5) was performed to test unadjusted associations between categorical variables in basic characteristics (Table 1), intraoperative variables (Table 2), pre- and intraoperative interventions (Table 3), postoperative compliance variables (Table 4), secondary outcomes and AL. Wilcoxon’s rank sum test was performed to test the unadjusted association between continuous variables (Table 1, 2, 3, 4 and LOS) and AL.

**Table 1:** Basic characteristics stratified by anastomotic leakage

	Anastomotic leakage			
	No (*N* = 1745)	Yes (*N* = 155)	Unadjusted analysis	Adjusted analysis
Sex			*χ*^2^ (1) = 15.08, *p* < 0.001	**1.91 (1.30, 2.79)**
Male	992 (56.9)	113 (72.9)		
Female (reference group)	753 (43.1)	42 (27.1)		
Missing	0 (0.0)	0 (0.0)		
Age (years)			*Z* = 3.64*, p* < 0.001	**0.98 (0.96, 0.99)**
*N*	1743	155		
Missing *N*	2 (0.1)	0 (0.0)		
Mean (SD)	68.09 (11.0)	64.99 (10.6)		
Year of operation			*χ*^2^ (1) = 12.81, *p* < 0.001	**1.84 (1.22, 2.79)**
2010–2015 (reference group)	645 (37.0)	35 (22.6)		
2016–2020	1100 (63.0)	120 (77.4)		
Missing	0 (0.0)	0 (0.0)		
Preoperative WHO score			*p* = 0.060	
Asymptomatic	1267 (72.6)	121 (78.1)		
Symptomatic but completely ambulatory	233 (13.4)	14 (9.0)		
Symptomatic, < 50% in bed during the day	25 (1.4)	0 (0.0)		
Symptomatic, > 50% in bed	2 (0.1)	1 (0.7)		
Missing	218 (12.5)	19 (12.2)		
Cancer			*p* = 0.237	
No	87 (5.0)	4 (2.6)		
Yes	1658 (95.0)	151 (97.4)		
Missing	0 (0.0)	0 (0.0)		
Preoperative nutritional treatment			*χ* ^2^ (1) = 0.58, *p* = 0.445	
No	1244 (71.3)	122 (78.7)		
Yes	190 (10.9)	15 (9.7)		
Missing	311 (17.8)	18 (11.6)		
Preoperative nutritional status			*p* = 0.464	
Normal status	1094 (62.7)	107 (69.0)		
Risk of malnutrition	205 (11.8)	15 (9.7)		
Malnourished	15 (0.8)	2 (1.3)		
Missing	431 (24.7)	31 (20.0)		
Smoking			*χ* ^2^ (2) = 3.71, *p* = 0.157	
No	1540 (88.2)	123 (79.3)		
Stopped due to surgery	53 (3.0)	8 (5.2)		
Yes	76 (4.4)	9 (5.8)		
Missing	76 (4.4)	15 (9.7)		
Alcohol			*χ* ^2^ (2) = 2.97, *p* = 0.226	
No	1027 (58.9)	90 (58.1)		
Stopped due to surgery	64 (3.7)	5 (3.2)		
Yes	55 (3.2)	9 (5.8)		
Missing	599 (34.2)	51 (32.9)		
Recreational drug use			*χ* ^2^ (1) = 2.87, *p* = 0.090	
No	1042 (59.7)	104 (67.1)		
Yes	28 (1.6)	6 (3.9)		
Missing	675 (38.7)	45 (29.0)		
Diabetes			*χ*^2^ (1) = 0.23, *p* = 0.633	
No	1505 (86.3)	131 (84.5)		
Yes	236 (13.5)	23 (14.8)		
Missing	4 (0.2)	1 (0.7)		
BMI (kg/m^2^)			*p* = 0.044	1.37 (0.30, 6.17) 1.16 (0.78, 1.72) **1.62 (1.01, 2.62)**
Under weight, 15 to < 18.5	28 (1.6)	2 (1.3)		
Normal weight (reference group), 18.5 to < 25	714 (40.9)	48 (31.0)		
Over weight, 25 to < 30	714 (40.9)	66 (42.6)		
Obese, $\ge$ 30	263 (15.1)	34 (21.9)		
Missing	26 (1.5)	5 (3.2)		
ASA physical status			*p* = 0.533	
1 (reference group)	331 (19.0)	36 (23.2)		
2	992 (56.9)	85 (54.8)		
3	383 (22.0)	31 (20.1)		
4	14 (0.8)	0 (0.0)		
5	1 (0.1)	0 (0.0)		
Missing	24 (1.4)	3 (1.9)		
Severe heart disease			*χ*^2^ (1) = 1.15, *p* = 0.284	
No	1128 (64.6)	114 (73.6)		
Yes	76 (4.4)	11 (7.1)		
Missing	541 (31.0)	30 (19.3)		
Severe pulmonary disease			*χ*^2^ (1) = 1.01, *p* = 0.314	
No	1176 (67.4)	120 (77.4)		
Yes	30 (1.7)	5 (3.2)		
Missing	539 (30.9)	30 (19.4)		
Preoperative chemotherapy			*χ*^2^ (1) = 0.004, *p* = 0.950	
No	1535 (88.0)	137 (88.4)		
Yes	205 (11.8)	18 (11.6)		
Missing	5 (0.2)	0 (0.0)		
Preoperative radiotherapy			*χ*^2^ (1) = 5.64, *p* = 0.018	1.35 (0.96, 1.91)
No (reference group)	1012 (58.0)	75 (48.4)		
Yes	726 (41.6)	80 (51.6)		
Missing	7 (0.4)	0 (0.0)		
Previous surgery to the abdominal region			*χ*^2^ (1) = 1.31, *p* = 0.252	
No	1297 (74.3)	123 (79.4)		
Yes	427 (24.5)	32 (20.6)		
Missing	21 (1.2)	0 (0.0)		
Preadmission stoma counselling			*χ*^2^ (1) = 0.03, *p* = 0.861	
No	128 (7.3)	14 (9.0)		
Yes	1050 (60.2)	109 (70.3)		
Missing	567 (32.5)	32 (20.7)		

Uni- and multivariate analysis

*χ*^2^ test or Fisher exact test (for categorical variables with cell size less than 5) was performed to test the unadjusted association between each categorical basic characteristic listed and anastomotic leakage. For difference in age, Wilcoxon’s rank sum test was performed

Values in parenthesis are percentages, except in the column for unadjusted associations, here values in parenthesis = degrees of freedom. ASA (American Society of Anesthesiologists physical status), BMI (body mass index)

Odds ratios and 95% confidence interval were reported for adjusted model which includes measures of, gender, age, body mass index, peritoneal soiling, preoperative radiotherapy, year of surgery, duration of primary surgery (hours) and preadmission patient education given (Table 3)

**Table 2:** Intraoperative variables stratified by anastomotic leakage

	Anastomotic leakage			
	No (*N* = 1745)	Yes (*N* = 155)	Unadjusted analysis	Adjusted analysis
Surgical procedure			*χ*^2^ (1) = 7.61, *p* = 0.022	
Robotic (reference group)	460 (26.3)	56 (36.1)		
Open	774 (44.4)	55 (35.5)		
Laparoscopic	511 (29.3)	44 (28.4)		
Missing	0 (0.0)	0 (0.0)		
Additional procedures			*χ*^2^ (1) = 0.46, *p* = 0.497	
No (reference group)	1141 (65.4)	117 (75.5)		
Yes	134 (7.7)	11 (7.1)		
Missing	470 (26.9)	27 (17.4)		
Peritoneal soiling/contamination			*χ*^2^ (1) = 9.22, *p* = 0.002	
No (reference group)	1566 (89.7)	121 (78.1)		
Yes	104 (6.0)	18 (11.6)		1.79 (1.02, 3.14)
Missing	75 (4.3)	16 (10.3)		
Systemic opioids given during surgery			*χ*^2^ (1) = 2.08, *p* = 0.149	
No	231 (13.2)	17 (11.0)		
Yes	976 (55.9)	106 (68.4)		
Missing	538 (30.9)	32 (20.6)		
Depth of anesthesia monitored			*χ*^2^ (1) = 1.59, *p* = 0.207	
No	343 (19.7)	28 (18.1)		
Yes	887 (50.8)	96 (61.9)		
Missing	515 (29.5)	31 (20.0)		
Pre- and intraoperative ERAS compliance rate (%)			*Z* = 0.82, *p* = 0.410	
N	1549 (88.8)	137 (88.4)		
Missing N	196 (11.2)	18 (11.6)		
Mean (SD)	93.15 (7.91)	92.66 (8.94)		
Duration of primary surgery (hours)			*Z* = −5.21, *p* < 0.001	1.13 (1.03, 1.23)
N	1732 (99.3)	151 (97.4)		
Missing N	13 (0.7)	4 (2.6)		
Mean (SD)	4.64 (1.69)	5.48 (2.22)		
New ileostomy			χ^2^ (1) = 5.23, *p* = 0.022	
No	739 (42.3)	51 (32.9)		
Yes	1006 (57.7)	104 (67.1)		
Missing	0 (0.0)	0 (0.0)		

Univariate and multivariate analysis

*χ*^2^ test or Fisher exact test (for categorical variables with cell size less than 5) was performed to test unadjusted association between each intraoperative variable and anastomotic leakage. For difference in ERAS compliance rate and duration of primary surgery, Wilcoxon’s rank sum test was performed

Values in parenthesis are percentages if not stated otherwise, except in the column for unadjusted associations, here values in parenthesis = degrees of freedom

Odds ratios and 95% confidence interval were reported for adjusted analysis which includes measures of, gender, age, body mass index, peritoneal soiling, preoperative radiotherapy, year of surgery, duration of surgery (hours) and preadmission patient education

**Table 3:** Preoperative and intraoperative compliance stratified by anastomotic leakage

	Anastomotic leakage		
	No (*N* = 1745)	Yes (*N* = 155)	Unadjusted association
Preoperative compliance			
Preadmission patient education given			*χ*^2^ (1) = 2.24, *p* = 0.134
Non-compliant	52 (3.0)	8 (5.2)	
Compliant	1687 (96.7)	146 (94.2)	
Missing	6 (0.3)	1 (0.6)	
Preoperative oral carbohydrate treatment			*χ*^2^ (1) = 2.03, *p* = 0.155
Non-compliant	79 (4.5)	11 (7.1)	
Compliant	1610 (92.3)	140 (90.3)	
Missing	56 (3.2)	4 (2.6)	
Mechanical bowel preparation			–
Non-compliant	0 (0.0)	0 (0.0)	
Compliant	387 (22.2)	21 (13.6)	
Missing	12 (0.7)	1 (0.6)	
Not applicable	1346 (77.1)	133 (85.8)	
Preoperative long-acting sedative medication			*χ*^2^ (1) = 0.08, *p* = 0.779
Non-compliant	240 (13.8)	22 (14.2)	
Compliant	1459 (83.6)	125 (80.6)	
Missing	46 (2.6)	8 (5.2)	
Antibiotic prophylaxis			0.394
Non-compliant	19 (1.1)	0 (0.0)	
Compliant	1721 (98.6)	153 (98.7)	
Missing	5 (0.3)	2 (1.3)	
Thrombosis prophylaxis			*χ*^2^ (1) = 0.02, *p* = 0.891
Non-compliant	64 (3.7)	6 (3.9)	
Compliant	1676 (96.0)	148 (95.5)	
Missing	5 (0.3)	1 (0.6)	
PONV prophylaxis administered			0.725
Non-compliant	43 (2.5)	1 (0.7)	
Compliant	738 (42.3)	43 (27.7)	
Missing	8 (0.5)	1 (0.6)	
Not applicable	956 (54.7)	110 (71.0)	
*Intraoperative compliance*			
Infusion of vasoactive drugs			*χ*^2^ (1) = 1.46, *p* = 0.227
Non-compliant	436 (25.0)	32 (20.7)	
Compliant	1242 (71.2)	117 (75.5)	
Missing	67 (3.8)	6 (3.8)	
Upper-body forced-air heating cover used			1.000
Non-compliant	46 (2.6)	4 (2.6)	
Compliant	1667 (95.5)	149 (96.1)	
Missing	32 (1.9)	2 (1.3)	
Total IV volume of fluids intraoperatively			*χ*^2^ (1) = 4.62, *p* = 0.032
Non-compliant	63 (3.6)	11 (7.1)	
Compliant	1682 (96.4)	144 (92.9)	
Missing	0 (0.0)	0 (0.0)	
Preoperative compliance rate (%)			*Z *= 0.97, *p* = 0.332
N	1617 (92.7)	142 (91.6)	
Missing N	128 (7.3)	13 (8.4)	
Mean (SD)	95.14 (9.04)	94.34 (10.18)	
Intraoperative compliance rate (%)			*Z* = −0.45, *p* = 0.651
*N*	1654 (94.8)	149 (96.1)	
Missing N	91 (5.2)	6 (3.9)	
Mean (SD)	89.16 (17.10)	89.49 (17.79)	

*χ*^2^ test or Fisher exact test (for categorical variables with cell size less than 5) was performed to test unadjusted association between each pre- and intra-operative compliance (compliant vs non-compliant) and anastomotic leakage. No Fisher exact test was calculated for oral bowel preparation since dichotomous responses were not present (the entire row for non-compliant was zero). For difference in compliance rate, Wilcoxon’s rank sum test was performed. Values in parenthesis are percentages if not stated otherwise, except in the column for unadjusted associations, here values in parenthesis = degrees of freedom

**Table 4:** Postoperative compliance stratified by anastomotic leakage

	Anastomotic leakage		
	No (*N* = 1745)	Yes (*N* = 155)	Unadjusted association
Opioid use—On day of surgery			*x*^2^(1) = 4.98, *p* = 0.026
Non-compliant	741 (42.5)	88 (56.8)	
Compliant	479 (27.4)	36 (23.2)	
Missing	525 (30.1)	31 (20.0)	
Opioid use—On POD 1			*x*^2^(1) = 15.84, *p* < 0.001
Non-compliant	687 (39.4)	93 (60.0)	
Compliant	529 (30.3)	31 (20.0)	
Missing	529 (30.3)	31 (20.0)	
Opioid use—On POD 2			*x*^2^(1) = 13.05, *p* < 0.001
Non-compliant	729 (41.8)	94 (60.7)	
Compliant	466 (26.7)	27 (17.4)	
Missing	550 (31.5)	34 (21.9)	
Opioid use—On POD 3			*x*^2^(1) = 14.87, *p* < 0.001
Non-compliant	710 (40.7)	96 (61.9)	
Compliant	431 (24.7)	24 (15.5)	
Missing	604 (34.6)	35 (22.6)	
Total IV volume of fluids day 0 (mL)			*Z* = −1.52, *p* = 0.129
*N*	1745	155	
Mean (SD)	2869.79 (1601.67)	3124.64 (1804.02)	
Missing	0 (0.0)	0 (0.0)	
Duration of IV fluid infusion (days)			*Z *= −2.83, *p* = 0.005
*N*	1701	148	
Mean (SD)	1.95 (4.02)	3.51 (6.46)	
Missing	44 (2.5)	7 (4.5)	
Time to passage of flatus (days)			*Z* = −1.47, *p* = 0.143
*N*	1453	117	
Mean (SD)	1.63 (1.85)	1.97 (2.18)	
Missing	292 (16.7)	38 (24.5)	
First passage of stool (days)			*Z* = −0.72, *p* = 0.473
*N*	1652	142	
Mean (SD)	2.42 (3.58)	3.09 (4.53)	
Missing	93 (5.33)	13 (8.39)	
Time to tolerating solid food (days)			*Z* = −5.18, *p* < 0.001
*N*	1546	122	
Mean (SD)	3.13 (4.78)	5.95 (7.25)	
Missing	199 (11.4)	33 (21.3)	
Termination of urinary drainage (days)			*Z *= −4.43, *p* < 0.001
*N*	1497	119	
Mean (SD)	4.51 (7.21)	7.84 (8.88)	
Missing	248 (14.2)	36 (23.2)	
Time to pain control with oral analgetics (days)			*Z* = −4.28, *p* < 0.001
*N*	1660	133	
Mean (SD)	3.63 (4.28)	5.44 (4.85)	
Missing	85 (4.87)	22 (14.19)	

*x*^2^ test or Fisher exact test (for categorical variables with cell size less than 5) was performed to test the difference in post-operative compliance for categorical variables, whereas, Wilcoxon’s rank sum test was performed for continuous variables

Values in parenthesis are percentages if not stated otherwise, except in the column for unadjusted associations, here values in parenthesis = degrees of freedom

Multivariate logistic regression was estimated based on purposeful selection modeling approach [17] from basic characteristics, intraoperative variables, and pre- and intraoperative compliance rate. Potential risk factors with *p* < 0.25 in the unadjusted models were included in the initial step of multivariate logistic regression; then risk factors with *p* > 0.1 and a change in any remaining parameter estimates < 15% compared to the full model (the initial step) were successively removed. Variables included in multivariable analyses had 0.1%–37.9% missing information. This was handled via multiple imputation using iterative chained equations [19].

Normal distribution was tested using Shapiro–Francia test. Compliance data were calculated as the numbers of achieved interventions divided with the total number of pre- and intraoperative interventions. To avoid bias, only compliance to pre- and intraoperative interventions was analyzed, since postoperative interventions could be considered as variables related to the outcome of surgery [20].

Conversion to open surgery was analyzed on intention-to-treat basis.

Categorical variables were presented as frequencies and percentage and continuous variables as mean with standard deviation (SD) or median with interquartile range when appropriate. Results from multivariate logistic analysis were reported as OR and 95% CI.

A *p* value < 0.05 or 95% CI not including 1 was considered statistically significant. Stata version 16.0 (StataCorp, College Station, Texas, United States of America) was used for statistical analysis.

## Results

In all, 1900 patients were included in the study, 155 (8.2%) with AL. Of all AL, 54 (34.8%) had a radiological diagnosis only, without intervention, 13 (8.4%) had a percutaneous drain only, and 88 (56.8%) had a reoperation.

### Time, leak rate and surgical approach

**Fig. 1:**
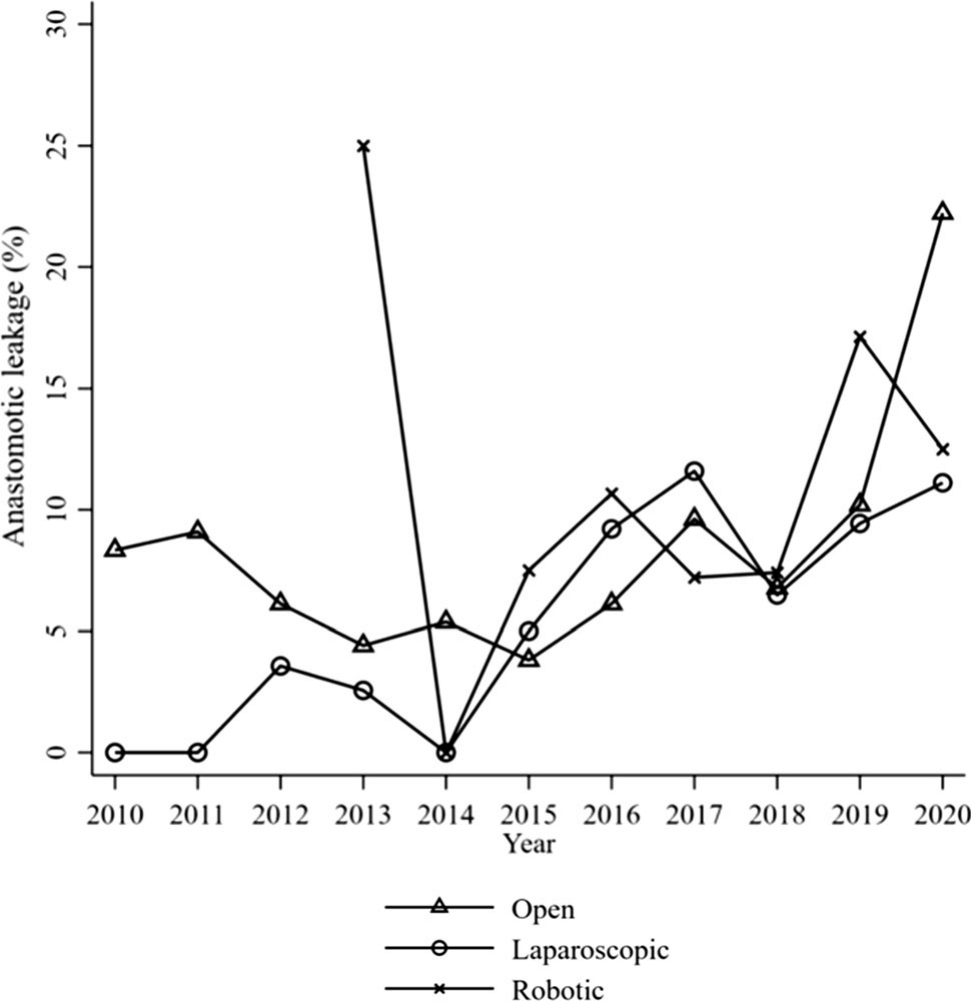
Anastomotic leakage over time stratified by surgical approach

Figure 1 illustrates AL rate over time stratified on surgical approach. In 2013, only 4 robotic operations were performed, and one suffered a leak. The frequency of AL increased over time for all modalities and surgery late in the study period (2016–2020) was a risk factor for AL (9.8% vs 5.1%, OR 1.84, 95% CI (1.22, 2.79)) compared to surgery early in the study period (2010–2015), demonstrated in Table 1.

### Basic characteristics and intraoperative variables

Basic characteristics stratified by AL are shown in Table 1. Males had a higher AL rate than females (10.2% vs 5.3%, OR 1.91, 95% CI (1.30, 2.79))**.** Obesity was a risk factor for AL (11.5% vs 6.3%, OR 1.62, 95% CI (1.01, 2.62)) compared to normal weight. Although preoperative radiotherapy was a significant risk factor for AL in univariate analysis, this association did not remain in the multivariate model, OR 1.29 (0.91, 1.83).

Patients with peritoneal contamination had a higher AL rate compared to patients without contamination (14.8% vs 7.2%, OR 1.79, 95% CI (1.02, 3.14)), as shown in Table 2. Duration of primary surgery and age were both associated with AL OR 1.13, 95% CI (1.03, 1.23) and OR 0.98, 95% CI (0.96, 0.99) respectively.

### Compliance to the ERAS protocol

Pre- and intraoperative compliance measures to the ERAS protocol are shown in Table 3. Comparing unadjusted AL rates in compliant vs non-compliant, one item–total iv volume of fluids intraoperatively, showed significant difference in favor of the compliant group (7.9% vs 14.9%, *χ*^2^ (1) = 4.62, *p* = 0.032); however, fluid management failed to reach significance in the multivariate logistic regression. The reverse was shown for preadmission patient education, which was not significant in univariate analysis but showed to be an independent protective factor for AL after adjustment (OR 0.41, 95% CI (0.19. 0.92). Overall, mean (SD) pre- and intraoperative compliance rates were high in both the AL group (92.66% (8.94)) and the no AL group (93.15% (7.91)) and showed no significant differences in unadjusted (Z = 0.82, *p* = 0.410) or adjusted (OR 0.99, 95% CI (0.97, 1.01)) analysis. Patients suffering from AL demonstrated significantly worse postoperative compliance (Table 4).

### Short-term outcome

**Fig. 2:**
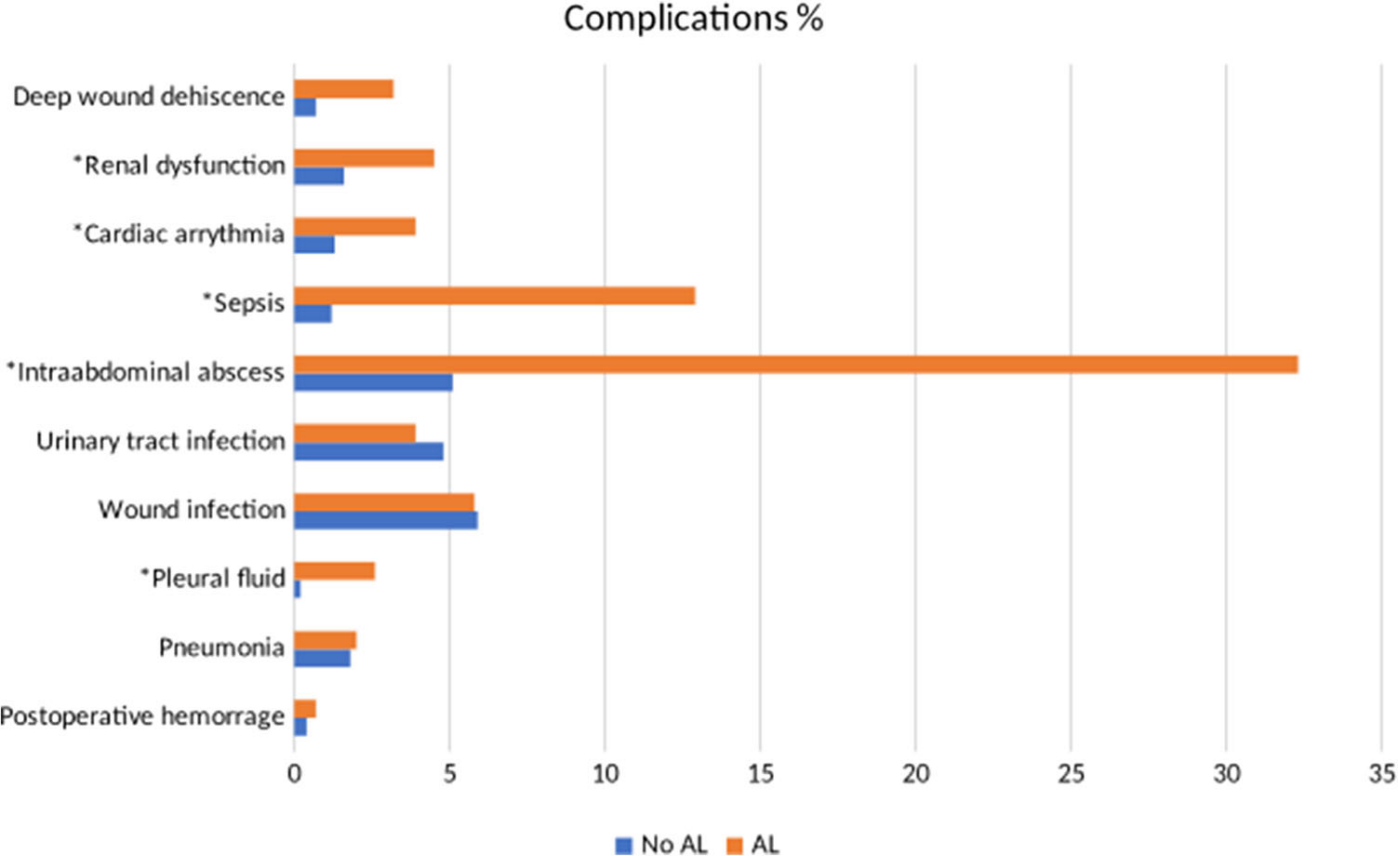
Selected complications (%) stratified by AL. *P value less than 0.05 was considered significant. Further 12 complications were compared (not shown) without significant difference between groups

Major complications (Clavien–Dindo ≥ 3) were more common in patients with AL (63.9% vs 5.9%, *p* < 0.001). Results favored the non-AL group regarding reoperations (6.6% vs 69.7%, *χ*^2^ (1) = 542.68, *p* < 0.001) and LOS (median 7 vs 15 nights, Z = −11.72, *p* < 0.001). No difference was observed in postoperative mortality (30 days). Selected complications stratified by AL are shown in Fig. 2.

## Discussion

In this large multicenter cohort study within an ERAS protocol, male gender, time of surgery, obesity, longer duration of surgery, peritoneal contamination, age and preadmission education were found to be independent predictors of anastomotic leakage in patients with rectal tumor operated with anterior resection. Overall compliance to pre- and intraoperative interventions in the ERAS protocol was high regardless of AL and did not affect the risk of AL. When evaluating single compliance items, total iv volume of fluids intraoperatively demonstrated significantly better compliance in patients without AL in univariate analysis. However, the variable did not qualify as an independent risk factor after adjusting for other covariates. After adjustment, only preadmission education remained as a protective ERAS compliance variable.

Results diverge in the literature on risk factors for AL [2, 3, 8, 21]. This may partly be explained by factors such as heterogeneity in patient recruitment, lack of description regarding perioperative treatment protocols differences in follow-up and underpowered studies. Furthermore, a lack of international consensus on how to define AL, with a vast variability in terminology and grading terms resulting in uncertainty on how to clinically diagnose AL (by symptoms, radiography or reoperation), makes a comparison between different studies difficult.

In 2010, guidelines defining and grading AL following rectal surgery was published [22] and later validated [23]. AL was in the guidelines defined as a “defect of the intestinal wall at the anastomotic site leading to a communication between the intra- and extraluminal compartments” and graded according to Grade A, Grade B and C depending on severity of AL.

Despite these efforts made, surgeons still have different opinions regarding definitions of AL and what actions are needed in order to treat AL.

The EIAS definition of AL is either radiological findings and/or a reoperation, to some extent contribute to the general variability between studies. However, the definition is identical for all centers recording in the database, and thus, all AL were recorded according to the same terms. AL rate in the current study was 8.2%, in line with previous results from literature where compliance to ERAS elements is unknown [2, 3]. The majority were leaks which required reoperation. However, since about 30% of AL are diagnosed beyond 30 days after surgery, the true leakage rate in the present study is likely to be underestimated [24].

The multivariate analysis identified male gender, obesity, peritoneal contamination, time of surgery, duration of surgery, age and preadmission education as independent predictors for AL. The majority of these predictors are previously reported, for example male gender due to a narrow pelvis, more difficult surgery [25, 26] or hormone-related differences compared to females impeding microcirculation [27]. The literature also supports the relationship between high BMI and AL [2, 3, 8], explained by more difficult surgery and a larger burden of comorbidities in obese patients. Intraoperative contamination is also a recognized risk factor for AL [28]. Operation time is another recognized risk factor for AL [2, 8]. Suggested causal factors such as obesity, adverse intraoperative events, increased bacterial exposure and hypoperfusion might explain this association.

The finding that surgery late in the study period was associated with an increased rate of AL is unexpected since studies have shown that leak rates seem to be stable over time [21, 29]. We did not find a clear explanation for this finding, but possible explanations are that diagnostics with X-ray investigations have increased over time, a more thorough registration of AL and increased use of neoadjuvant radiotherapy later in the study period. Older age was identified as a protective factor against AL in our study, not in line with data from studies showing results in the opposite direction [30, 31]. This discrepancy may be by chance since the effect, although statistically significant, was marginal. Preadmission education has never before been investigated in AL research. Previous studies show a possible impact on variables such as pain and anxiety [32, 33], but the association with AL in this study is more difficult to explain. Education might help the patients improve their conditions before surgery.

Other previously presented risk factors for AL such as smoking/alcohol consumption [34, 35], diabetes [36], pulmonary disease [37], high ASA fitness grade [38], poor preoperative nutritional status measured as weight loss or hypoalbuminemia [39, 40], preoperative chemotherapy or preoperative radiotherapy [9, 25, 41] could not be identified as risk factors for AL in the current multivariate analysis.

Neither surgical approach (open, laparoscopic, robotic) nor conversion to open surgery were found to be risk factors for AL, the latter somewhat surprising since conversion have been described as a proxy for difficult surgery [42] and reported to result in higher complication rates [43], but not AL per se.

AL was, as expected, associated with increased short-term morbidity, higher rate of reoperations and longer LOS. Our study did not demonstrate a difference in mortality between groups, but mortality may rise over 90 days and this may better reflect the true mortality related to the operation [7].

Even though increased compliance to the ERAS protocol has been shown to improve short-term outcome in previous studies [20], comparing pre- and intraoperative compliance in patients with and without AL in the current study revealed one care item—excess of IV volume of fluids intraoperatively, as a univariate predictor for AL. Perioperative overhydration has been associated with poor outcome and even increased risk for AL in studies [44, 45], although underhydration might also confer increased morbidity [46]. The finding that no other compliance item made a significant difference in univariate analysis may well be explained by the overall high compliance in both groups. Postoperative compliance measures, i.e., variables related to outcome from surgery, showed—as expected—worse results in the AL group.

The strength of the current study is the multicenter design, collecting over 300 prospectively and consecutively recorded variables from the ERAS database which makes it possible to study multiple important perioperative covariates that may to a larger extent reflect clinical reality compared to data collected in randomized trials. Also, by measuring compliance, we can control for the treatment before and during surgery.

On the other hand, the retrospective design makes it difficult to draw firm conclusions on outcome, and although we tried to minimize errors using multivariate analysis, unknown factors affecting the results cannot be ruled out. Such a limitation is time. During the 10-year long inclusion period, changes can occur in attitudes and turnover among staff in the ERAS program as well as technical progress. Despite that time was adjusted for in the multivariate analysis and compliance to the protocol was similar between groups over time, this time factor might be difficult to fully eliminate. A weakness in trying to study the role of compliance to ERAS as risk factor for AL is the uniform and high compliance to the protocol which reduce chances of detecting such a role. Another weakness is the lack of data on tumor height and level of anastomosis in EIAS, factors that are important for the risk of developing AL. Other drawbacks acknowledged are limitations in the register itself that could introduce bias, such as short follow-up time (30 days) and lack of data on postoperative pathology, e.g. staging.

In conclusion, in this large multicenter cohort study in hospitals with high compliance to an ERAS program, we found seven independent risk factors for AL: male gender, high BMI, year of surgery, peritoneal contamination, duration of surgery, age and preadmission education in patients operated on with anterior resection. The only protective compliance item remaining after adjustment for covariates was preadmission education. The absence of impact from overall compliance may in part be explained by high and uniform ERAS compliance in the participating centers of the study. Further studies should aim at preparing patients within an optimization program several weeks before major surgery, taking risk factors for AL into account.
